# Untargeted metabolomics analysis reveals key pathways responsible for the synergistic killing of colistin and doripenem combination against *Acinetobacter baumannii*

**DOI:** 10.1038/srep45527

**Published:** 2017-03-30

**Authors:** Mohd Hafidz Mahamad Maifiah, Darren J. Creek, Roger L. Nation, Alan Forrest, Brian T. Tsuji, Tony Velkov, Jian Li

**Affiliations:** 1 Drug Delivery, Disposition and Dynamics, Monash Institute of Pharmaceutical Sciences, Monash University, Parkville, VIC, 3052, Australia; 2 UNC Eshelman School of Pharmacy, The University of North Carolina at Chapel Hill, Chapel Hill, NC, 27599-7569, USA; 3 Department of Pharmacy Practice, University at Buffalo, Buffalo, NY, USA; 4 Monash Biomedicine Discovery Institute, Department of Microbiology, Monash University, VIC 3800, Australia

## Abstract

Combination therapy is deployed for the treatment of multidrug-resistant *Acinetobacter baumannii*, as it can rapidly develop resistance to current antibiotics. This is the first study to investigate the synergistic effect of colistin/doripenem combination on the metabolome of *A. baumannii*. The metabolite levels were measured using LC-MS following treatment with colistin (2 mg/L) or doripenem (25 mg/L) alone, and their combination at 15 min, 1 hr and 4 hr (n = 4). Colistin caused early (15 min and 1 hr) disruption of the bacterial outer membrane and cell wall, as demonstrated by perturbation of glycerophospholipids and fatty acids. Concentrations of peptidoglycan biosynthesis metabolites decreased at 4 hr by doripenem alone, reflecting its mechanism of action. The combination induced significant changes to more key metabolic pathways relative to either monotherapy. Down-regulation of cell wall biosynthesis (via D-sedoheptulose 7-phosphate) and nucleotide metabolism (via D-ribose 5-phosphate) was associated with perturbations in the pentose phosphate pathway induced initially by colistin (15 min and 1 hr) and later by doripenem (4 hr). We discovered that the combination synergistically killed *A. baumannii* via time-dependent inhibition of different key metabolic pathways. Our study highlights the significant potential of systems pharmacology in elucidating the mechanism of synergy and optimizing antibiotic pharmacokinetics/pharmacodynamics.

Multidrug-resistant (MDR) *Acinetobacter baumannii* has been classified by the Centers for Diseases Control and Prevention (CDC) as a ‘‘Serious Threat’’ which is responsible for a plethora of nosocomial infections including pneumonia, bacteraemia, wound infections, urinary tract infections and meningitis^1^,^2^,^3^. As one of the six significant ESKAPE ‘superbugs’ identified by the Infectious Diseases Society of America (IDSA), *A. baumannii* represents a challenge as it can rapidly develop resistance to all clinically available antibiotics^4^,^5^,^6^,^7^. *A. baumannii* exhibits a wide array of antibiotic resistance strategies, including degradation and modification of enzymes, alteration of target binding sites, and activation of efflux pumps^8^.

Due to the dry antibiotic discovery pipeline, the re-utilization of the ‘old’ polymyxin class of antibiotics has become essential for the treatment of life-threatening infections caused by MDR *A. baumannii*^9^. Polymyxin B and colistin (i.e. polymyxin E) are non-ribosomal cyclic lipopeptides that contain six basic l-α-γ-diaminobutyric acid (Dab) residues, two hydrophobic amino acids, and an *N*-terminal fatty acyl group^10^. Polymyxins interact electrostatically with the phosphate groups of the lipid A component of lipopolysaccharide (LPS) followed by non-polar interactions of hydrophobic domains on both molecules to initiate the rapid bactericidal effect^10^,^11^. Destabilization of the LPS leaflet of the outer membrane has generally been thought to cause local disturbance, osmotic imbalance and finally cell death, although the ultimate mechanism of cell death is not completely understood^12^. Polymyxin monotherapy may lead to treatment failure as it is not always possible to generate reliably efficacious plasma exposure and bacterial resistance may emerge^13^,^14^,^15^,^16^,^17^,^18^. *A. baumannii* can become resistant to polymyxins by the addition of phosphoethanolamine (pEtN), galactosamine (GalN) or both^19^,^20^,^21^,^22^,^23^,^24^ to its lipid A structure, or by the loss of LPS^25^. These modifications significantly reduce the negative charge on the bacterial outer membrane, thus diminishing the binding of polymyxins^10^. A number of *in vitro* studies have shown that colistin and doripenem combination therapy is synergistic against MDR *Pseudomonas aeruginosa, Klebsiella pneumoniae* and *A. baumannii*^26^,^27^,^28^,^29^,^30^,^31^. In addition, the colistin-carbapenem combination has been shown to significantly limit the emergence of colistin resistance in *A. baumannii*^32^. Therefore, polymyxin-carbapenem combinations are often employed to enhance therapeutic response and minimize potential polymyxin resistance.

The mechanisms that underlie the synergistic action of polymyxins and carbapenems have not been fully elucidated. Metabolomics provides the opportunity to gain a system-wide snapshot of cellular biochemical networks under defined conditions^33^,^34^,^35^, and has been increasingly employed in bacterial physiology^34^ and drug discovery to elucidate the mechanism of drug action^36^. Furthermore, a detailed understanding of cellular metabolic perturbations in response to antibiotic treatment can potentially facilitate the discovery of novel alternative drug targets^37^. To elucidate the mechanism of synergistic killing of the colistin and doripenem combination against *A. baumannii*, we conducted an untargeted metabolomics study. Our study is the first to reveal that the metabolic perturbations induced by the combination were predominantly associated with the effect of colistin in the early time points, followed by doripenem at 4 hr. Notably, significant metabolic changes via disorganization of membrane lipids and depletion of nucleotides, energy, and amino sugar metabolites were evident following treatment with colistin alone, and were enhanced by its combination with doripenem. Our data provide a novel insight into the mechanism of synergistic killing against *A. baumannii* by the colistin-doripenem combination.

## Results

**Figure 1:**
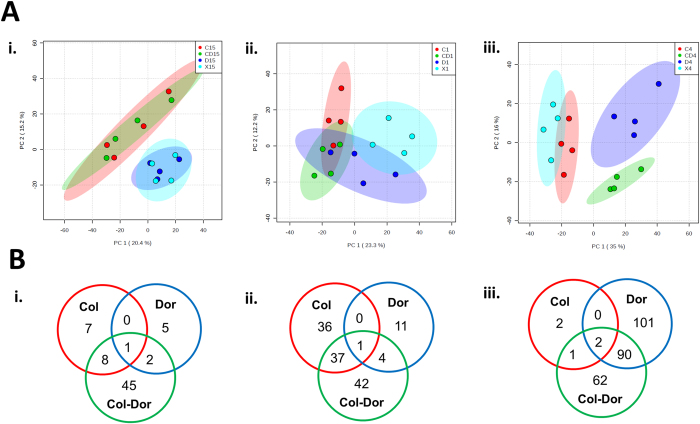
Multivariate and univariate analyses of global metabolic changes. (**A**) PCA score plots of the first two principal components for metabolite levels from samples treated with colistin, doripenem and the combination at (i) 15 min, (ii) 1 hr, and (iii) 4 hr. Each data set represents a total of 16 samples of 4 biological replicates of each condition. Red = colistin alone (C); Dark blue = doripenem alone (D); Green = colistin and doripenem combination (CD); Light blue = untreated control (X). (**B**) Venn diagrams represent the number of metabolites significantly affected by each treatment at (i) 15 min, (ii) 1 hr, and (iii) 4 hr. Significant metabolites were selected with ≥1.5-log_2_-fold, *p* ≤ 0.05, FDR ≤ 0.1 (one-way ANOVA for multiple comparison).

Untargeted metabolomics was applied to profile the metabolic changes in *A. baumannii* ATCC 19606 treated with monotherapy of colistin and doripenem and the combination at 15 min, 1 hr and 4 hr. Four biological replicates were independently prepared from different cultures on separate days, and all the samples were analyzed in a single LC-MS batch. The within-experiment technical (analytical) variations were monitored based on periodic analysis of pooled biological quality control (PBQC) samples in the batch. We showed that the median relative standard deviation (RSD) of the PBQC, an indicator for analytical reproducibility, was 14% (Supplementary Figure S1A) which is well within the acceptable limits for metabolomics^38^. In addition, the PCA plot showed the PBQC samples tightly clustered together, indicating minimal technical variation (Supplementary Figure S1B). The median RSD value for each sample group was between 19–30%, showing the dynamics of bacterial metabolism due to antibiotic treatments (Supplementary Figure S1A). Principal component analysis (PCA) (Fig. 1A) and heatmaps (Supplementary Figure S2) revealed global metabolic changes in *A. baumannii* after antibiotic treatment at each time point. A total of 1,577, 1,583 and 1,637 unique metabolites (carbohydrates, energy, amino acids, nucleotides, lipids, peptides, and others) were putatively identified at 15 min, 1 hr and 4 hr, respectively. Univariate analysis of these features revealed that 5–11% of metabolites were significantly altered (≥1.5-log_2_-fold; ANOVA, *p* ≤ 0.05, FDR ≤ 0.1) following treatment with monotherapy and the combination at each time point (Fig. 1B, and Supplementary Tables 1 and 2).

Colistin induced significant global metabolic changes as early as at 15 min. In contrast, the most substantial metabolic changes associated with doripenem monotherapy were observed at 4 hr, signifying the time-dependent effect of doripenem. Treatment with the colistin and doripenem combination affected 31 additional metabolites that were not altered by either colistin or doripenem treatment alone at 15 min and 1 hr, indicating a synergistic effect of this combination. Interestingly, the PCA plot (Fig. 1A) and heatmaps (Supplementary Figure S2) show relatively similar metabolic profiles between the treatment with colistin monotherapy and the combination of colistin and doripenem at 15 min. There was also considerable overlap at 1 hr as almost half of the perturbed metabolites from the combination treatment were also perturbed by colistin alone. However, at 4 hr the impact of colistin alone was minimal and the combination treatment shared many metabolic features with the doripenem monotherapy (Fig. 1B).

### Colistin alone and in combination with doripenem predominantly induced disruption of bacterial lipids

**Figure 2:**
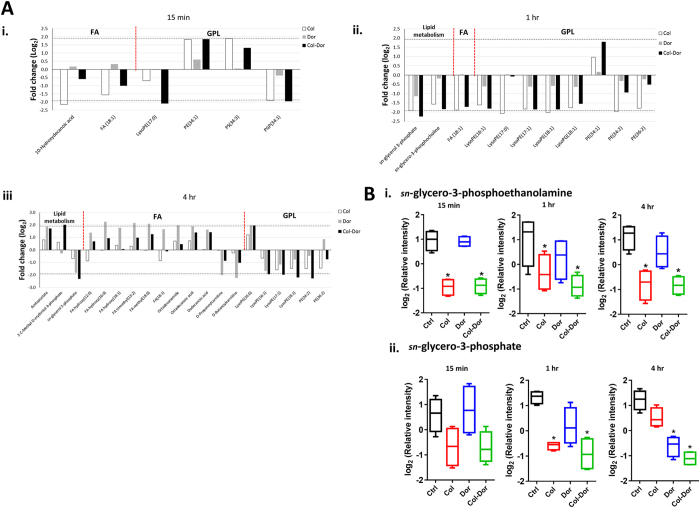
Perturbations of bacterial lipids. (**A**) Significantly perturbed lipids in *A. baumannii* ATCC 19606 following treatment with colistin (Col, white), doripenem (Dor, grey) and the combination (Col-Dor, black) for (i) 15 min, (ii) 1 hr, and (iii) 4 hr. Lipid names are putatively assigned based on accurate mass. (**B**) Depletion of (i) *sn*-glycero-3-phosphoethanolamine, and (ii) *sn*-glycero-3-phosphate after treatment with colistin, doripenem, and the combination across all three time points. Box plots indicate upper and lower quartiles (top and bottom of box); median (line within box); and the spread of data that are not outliers (whiskers). *≥1.5-log_2_-fold, *p *≤ 0.05, FDR ≤ 0.1 (one-way ANOVA).

Unique patterns of changes in the levels of lipids were observed in samples treated with either colistin monotherapy or combination with doripenem at 15 min, 1 hr and 4 hr. Treatment with colistin alone induced significant perturbation in the levels of membrane lipids at 15 min and 1 hr, predominantly the glycerophospholipids (GPLs) and fatty acids (FAs) (≥1.5-log_2_-fold; ANOVA, *p* ≤ 0.05, FDR ≤ 0.1) (Fig. 2A). Significant changes in levels of GPLs were observed after treatment with colistin and doripenem combination at all three time points, including the depletion of several lysophosphatidylethanolamines (lysoPE) while only very few FAs were affected. Interestingly, the metabolite arising from PE metabolism, *sn*-glycero-3-phosphoethanolamine, significantly decreased (≥1.5-log_2_-fold; ANOVA, *p* ≤ 0.0001, FDR ≤ 0.1) after treatment with colistin monotherapy and combination across all the time points (Fig. 2B). In addition, the combination therapy significantly decreased the level of *sn*-glycero-3-phosphate (≥1.5-log_2_-fold; ANOVA, *p* ≤ 0.001, FDR ≤ 0.1), another metabolite associated with GPL metabolism (Fig. 2B). Doripenem alone showed no significant changes to lipid levels at 15 min and 1 hr. However, doripenem alone caused substantial perturbation in the levels of cellular lipids, predominantly accumulation of FAs at 4 hr.

### Combination of colistin and doripenem induced global metabolic changes via Pentose Phosphate Pathway (PPP) metabolism

**Figure 3:**
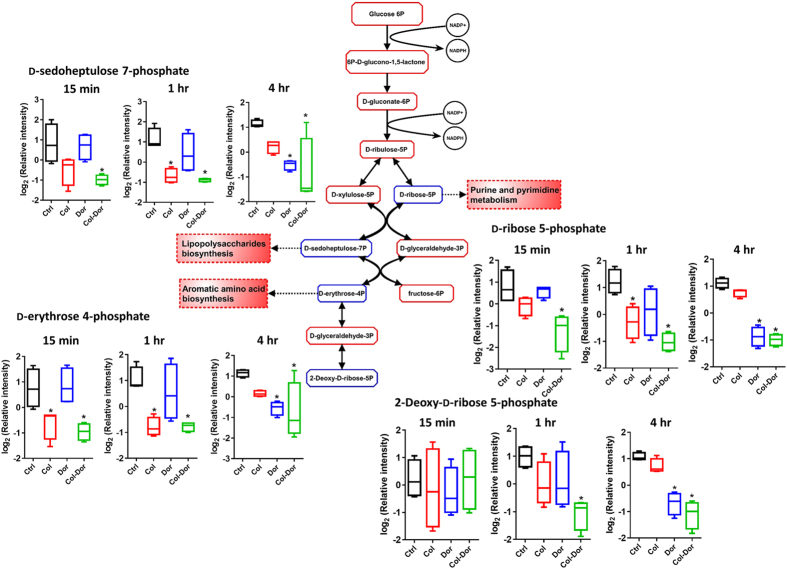
Central metabolic changes in the pentose phosphate pathway (PPP). Antibiotic treatment of *A. baumannii* ATCC 19606 significantly decreased the levels of three PPP metabolites (D-sedoheptulose 7-phosphate, D-erythrose 4-phosphate, and D-ribose 5-phosphate) that are essential anabolic precursors of related pathways. The combined colistin/doripenem significantly decreased the levels of the three precursor metabolites at all the time points. Additionally, 2-deoxy-D-ribose 5-phosphate significantly decreased followed by the combination at 1 hr and 4 hr. In the pathway flow chart (adapted from biocyc.org with reference to *E. coli* K-12), blue boxes indicate the metabolites that were significantly decreased and red boxes indicate the metabolites that were not significantly changed. Box plots indicate upper and lower quartiles (top and bottom of box); median (line within box); and the spread of data that are not outliers (whiskers). *≥1.5-log_2_-fold, *p* ≤ 0.05, FDR ≤ 0.1 (one-way ANOVA).

The combination of colistin and doripenem caused significant decreases in the levels of metabolites of central carbon metabolism, primarily associated with bacterial anabolic metabolism of the PPP at 15 min, 1 hr and 4 hr (≥1.5-log_2_-fold; ANOVA, *p* ≤ 0.001, FDR ≤ 0.1) (Fig. 3). In particular, the combination of colistin and doripenem induced significant decreases in the levels of three essential metabolites of PPP at all time-points, D-ribose 5-phosphate, D-sedoheptulose 7-phosphate, and D-erythrose 4-phosphate, key precursors for biosynthesis of nucleotides, lipopolysaccharides (LPS) and aromatic amino acids, respectively. These metabolites were depleted by colistin monotherapy at early time-points, but not by doripenem (significant at 1 hr); whereas significant depletion at 4 hr was observed for doripenem monotherapy, but not colistin. In addition to these PPP metabolites, a related metabolite, 2-deoxy-D-ribose-5-phosphate was consistently decreased as a result of the combination of colistin and doripenem at 1 hr and 4 hr.

### Colistin and doripenem caused depletion of metabolite levels of energy and nucleotide metabolism

**Figure 4:**
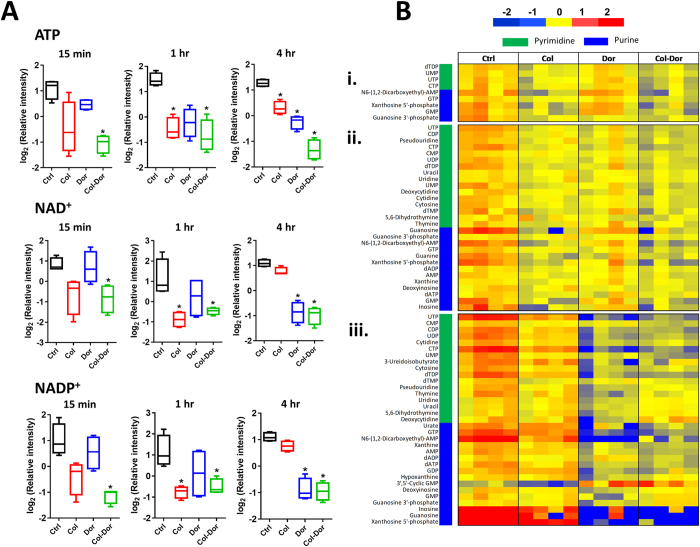
Depletion of energy and nucleotide metabolite levels. (**A**) Decreased levels of key energy-associated metabolites, ATP, NAD^+^ and NADP^+^ induced by colistin, doripenem, and the combination in *A. baumannii* ATCC 19606. Box plots indicate upper and lower quartiles (top and bottom of box); median (line within box); and the spread of data that are not outliers (whiskers). *≥1.5-log_2_-fold, *p* ≤ 0.05, FDR ≤ 0.1 (one-way ANOVA). (**B**) Heatmap profile of relative abundance of significantly perturbed nucleotides at (i) 15 min, (ii) 1 hr, and (iii) 4 hr after treatment with colistin (Col), doripenem (Dor) and the combination (Col-Dor) (n = 4). Antibiotics decreased the levels of nucleotides, both purines and pyrimidines, in *A. baumannii* ATCC 19606.

Significant depletion in the levels of intracellular metabolites of energy metabolism, namely ATP, NAD^+^ and NADP^+^, was observed following treatment with colistin and doripenem combination across all three time points (≥1.5-log_2_-fold; ANOVA, *p* ≤ 0.01, FDR ≤ 0.1) (Fig. 4A). Treatment with colistin alone decreased the levels of these energy metabolites at 15 min and 1 hr, while doripenem-associated depletion was only significant at 4 hr. Notably, significant perturbations of tricarboxylic acid (TCA) cycle intermediates, fumarate and cis-aconitate were identified in samples treated with colistin and doripenem alone and in combination in particular at 15 min and 4 hr (Supplementary Table 1). In addition, significant depletion in the levels of nucleotides, both purines and pyrimidines, were observed after colistin alone at 1 hr, doripenem alone at 4 hr and combination treatment at each time point (≥1.5-log_2_-fold; ANOVA, *p* ≤ 0.01, FDR ≤ 0.1) (Fig. 4B).

### Colistin and doripenem induced depletion of amino sugar metabolites for cell wall biosynthesis

**Figure 5:**
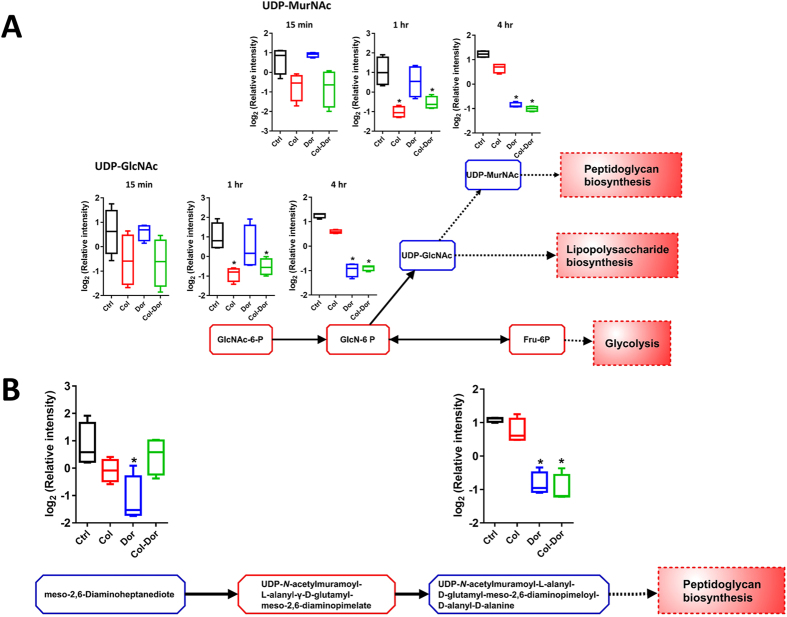
Depletion of amino sugar metabolites for peptidoglycan and lipopolysaccharide biosynthesis. (**A**) Significant decrease in the levels of two amino sugar metabolites at 1 hr and 4 hr by colistin, doripenem and the combination, and perturbation of the cell envelope biosynthesis (peptidoglycan and lipopolysaccharide biosynthesis) in *A. baumannii* ATCC 19606. UDP-*N*-acetyl-D-glucosamine (UDP-GlcNAc) is a key precursor metabolite for LPS and peptidoglycan biosynthesis. (**B**) Levels of two key metabolites of peptidoglycan biosynthesis significantly decreased after treatment with doripenem alone at 4 hr. The combination of colistin and doripenem also significantly decreased UDP-*N*-acetylmuramoyl-L-alanyl-D-glutamyl-meso-2,6-diaminopimeloyl-D-alanyl-D-alanine (>2.0-log_2_-fold) at 4 hr. The blue boxes in the flow charts indicate the metabolites that were significantly decreased. The red boxes indicate the metabolites that were not significantly changed. Box plots indicate upper and lower quartiles (top and bottom of box); median (line within box); and the spread of data that are not outliers (whiskers). *≥1.5-log_2_-fold, *p* ≤ 0.05, FDR ≤ 0.1 (one-way ANOVA).

Colistin alone significantly decreased the intracellular levels of several important metabolites associated with amino sugar and nucleotide sugar metabolism, in particular at 1 hr (≥1.5-log_2_-fold; ANOVA *p* ≤ 0.05, FDR ≤ 0.1) (Fig. 5A). The levels of two major precursor metabolites of cell wall biosynthesis significantly decreased after treatment with colistin alone at 1 hr, namely UDP-*N*-acetylmuramate (UDP-MurNAc) and UDP-*N*-acetylglucosamine (UDP-GlcNAc) (≥1.5-log_2_-fold; ANOVA *p* ≤ 0.01, FDR ≤ 0.1). Significant decreases in the levels of both metabolites were also observed following treatment with the combination of colistin and doripenem at 1 hr and 4 hr. Doripenem alone significantly decreased the amino sugar associated metabolites only at 4 hr. In addition to these metabolites, another two metabolites of peptidoglycan biosynthesis were identified to significantly decrease at 4 hr after doripenem treatment, meso-2,6-Diaminoheptanedioate and UDP-*N*-acetylmuramoyl-L-alanyl-D-glutamyl-6-carboxy-L-lysyl-D-alanyl-D-alanine (≥1.5-log_2_-fold; ANOVA *p* ≤ 0.01, FDR ≤ 0.1). Only UDP-*N*-acetylmuramoyl-L-alanyl-D-glutamyl-6-carboxy-L-lysyl-D-alanyl-D-alanine was found to significantly decrease after treatment with combined colistin/doripenem at 4 hr.

### Colistin and doripenem induced alterations in peptide metabolism

Treatment with doripenem alone and the combination of colistin and doripenem showed unique changes in the levels of short peptides (Supplementary Figure S3 and Tables 1,2,3). The number of significantly perturbed peptides increased across the time points after treatment with doripenem alone and the combination of colistin and doripenem (≥1.5-log_2_-fold; ANOVA *p* ≤ 0.05, FDR ≤ 0.1). However, colistin alone showed significant changes in the levels of only a few cellular peptides. Interestingly, a unique putative metabolite, tyramine (*m/z* = 137.08, t_R_ = 9.03 min; MSI level 2), which is associated with tyrosine metabolism was found to significantly increase only after treatment with doripenem alone and the combination of colistin and doripenem across all the time points (≥1.5-log_2_-fold; ANOVA *p* ≤ 0.0001, FDR ≤ 0.1).

## Discussion

The global spread of MDR Gram-negative bacteria is alarming and it is crucial to understand the detailed mechanisms of antibiotic action and resistance. Bacterial metabolic responses to antibiotics have not been well examined with cutting-edge metabolomics, and deciphering the metabolome of bacterial cells can potentially lead to innovative strategies for effective antibacterial therapy. Polymyxins and carbapenems display their primary antibacterial activity via initial interactions with LPS and binding to penicillin-binding proteins (PBPs), respectively^10^,^39^. Notwithstanding, increasing evidence indicates that the rarely explored effects on bacterial metabolism are crucial for the antibacterial activity of antibiotics^40^. The combination of polymyxins with carbapenems has been shown to be synergistic against MDR Gram-negative bacteria; albeit, the detailed mechanism of their synergistic action(s) has not been examined^26^,^27^,^28^,^29^,^30^. Previously, our transcriptomics data revealed that the combination of colistin and doripenem altered the gene expression profiles in *A. baumannii* at 1 hr in a similar manner to that of colistin treatment alone. These genes were primarily associated with outer membrane biogenesis, fatty acid metabolism and phospholipid trafficking^41^. Interestingly, similar transcriptional changes were also observed in the *A. baumannii* LPS-deficient strain without colistin treatment^42^. Our present study is the first to elucidate the synergistic killing mechanism of the combination of colistin and doripenem against *A. baumannii*. The most significant findings on the synergistic combination in this metabolomics study include: (1) differential time-dependent inhibition of key metabolic pathways; (2) perturbation of the PPP and the downstream metabolism of LPS and nucleotides; and (3) inhibition of cell wall synthesis via different targets.

In the present study, global metabolic changes of MDR *A. baumannii* were investigated following exposure to colistin and doripenem individually and in combination over 4 hr. Our results show, for the first time, that colistin, doripenem and the combination induced common global metabolic perturbations in *A. baumannii*, and metabolisms of cellular lipids, nucleotides, amino sugars and energy are common pathways involved in the synergistic action of colistin and doripenem (Figs 2, 3, 4, 5). The initial cellular metabolic perturbations following treatment with colistin monotherapy at 15 min and 1 hr impacted several essential metabolic pathways, namely lipid metabolism, nucleotide metabolism, amino sugar metabolism and energy metabolism (Figs 2, 4, and 5). Similar metabolic alterations were observed following treatment with doripenem alone at 4 hr (Figs 2, 4, and 5), indicating the effects of each antibiotic occur in a differential time-dependent manner. With the combination treatment, the perturbations were observed across all of the time points. This mechanistic finding has important implications for the pharmacokinetics/pharmacodynamics (PK/PD) of the colistin and doripenem combination, supporting its use in the clinic for maintaining persistent antibacterial effect and minimizing the potential bacterial regrowth due to colistin monotherapy^43^,^44^.

Two key models have been proposed to explain mechanisms of drug synergism, the parallel pathway inhibition model and the bioavailability model^45^,^46^,^47^,^48^. The parallel pathway inhibition model suggests that two drugs are synergistic if they inhibit two different targets in parallel pathways that are essential for an observed phenotype^47^. The bioavailability model suggests that two drugs are synergistic if one drug’s action enhances another drug’s availability in the target cell, either by increasing the second drug’s entry into the cell or by decreasing the second drug’s degradation or efflux^48^. As doripenem itself can access its target in the periplasmic space in *A. baumannii*, the bioavailability model is unlikely to explain the synergistic activity of colistin and doripenem, and is not supported by our metabolomics data. Our metabolomics analysis indicates that the parallel pathway inhibition model explains well the synergistic killing by colistin and doripenem against *A. baumannii*. Notably, treatment with colistin or doripenem alone or in combination at different time points significantly decreased the cellular levels of PPP intermediates (e.g. D-sedoheptulose 7-phosphate), UDP-GlcNAc and UDP-MurNAc, which are key precursor metabolites for the biosynthesis of peptidoglycan and LPS (Figs 3 and 5). Our metabolomics data also demonstrate that colistin and doripenem perturb various key pathways related to cell envelope biosynthesis, namely GPLs, FAs, LPS and peptidoglycan biosynthesis (Figs 2 and 5). Importantly, our study is the first to reveal that colistin itself also caused inhibition of cell wall synthesis by decreasing the essential precursor metabolites (i.e. UDP-GlcNAc and UDP-MurNAc), a different mechanism from doripenem which acts via binding to PBPs.

The Gram-negative bacterial cell envelope is composed of an asymmetrical outer membrane (OM), a thin cell wall, and a symmetrical inner membrane^49^. The outer leaflet of the OM is predominantly constituted of LPS and the inner leaflet is mainly comprised of phospholipids^49^,^50^. In line with the primary mode of action of colistin, our metabolomics data revealed that colistin treatment at 15 min and 1 hr caused significant perturbations in the levels of OM lipids, specifically GPLs and FAs (Fig. 2A). In keeping with this finding, our previous transcriptomics results showed that colistin treatment up-regulated the expression of the Mla system (Maintenance of OM lipid asymmetry) in *A. baumannii* ATCC 19606, which is responsible for transporting excess phospholipids in the outer leaflet back to the inner membrane to maintain the OM asymmetry^41^,^51^,^52^. Significant changes to the OM lipids, as observed at both the transcriptomics and metabolomics levels, are highly consistent with the proposed bactericidal mechanism of colistin via lipid exchange between the inner and outer membrane^11^. Furthermore, our previous transcriptomics data showed that colistin treatment induced the up-regulation of genes involved in fatty acid β-oxidation/degradation and down-regulation of genes involved in fatty acid biosynthesis^41^, which well explains the colistin-induced fatty acid perturbations observed here (Fig. 2A). Notably, doripenem treatment at 15 min and 1 hr did not produce any appreciable changes in the levels of GPLs and FAs relative to the untreated control (Fig. 2A), and the expression of lipid metabolism genes was not affected at 15 min, although significant transcriptomic changes were reported for doripenem treatment at 1 hr (i.e. retrograde phospholipid transport and lipoprotein transport)^41^. However, doripenem treatment at 4 hr produced a similar pattern of lipid changes (both GPLs and FAs) as per the aforementioned colistin treatment at 15 min and 1 hr. Interestingly, the entire time-course of the combination treatment displayed a distinct pattern of lipid changes, wherein only the GPLs were significantly perturbed while the FA levels remained largely unaffected. One metabolite involved in glycerophospholipid metabolism, s*n*-glycero-3-phosphoethanolamine, was specifically associated with colistin treatment, both alone and in combination, but s*n*-glycero-3-phosphoethanolamine was also significantly depleted in the LPS-deficient polymyxin-resistant strain *A. baumannii* 19606 R relative to the wild-type ATCC 19606 strain in the absence of polymyxin treatment^53^.

In terms of the impact on energy metabolism, treatment with the colistin/doripenem combination significantly decreased intracellular ATP, NADP^+^ and NAD^+^ levels and the levels of three major metabolites of PPP, namely D-sedoheptulose-7-phosphate, D-ribose 5-phosphate and D-erythrose 4-phosphate. ADP-heptose, a key downstream metabolite of the heptose biosynthesis pathway, is an important component of the LPS inner core^54^,^55^. Mutations in the gene (*GmhA*) associated with ADP-glyceromannoheptose synthesis in *Haemophilus influenza,* which cause deficiencies in heptose biosynthesis, result in an avirulent phenotype, increased membrane permeability and increased susceptibility to antibiotics^55^,^56^,^57^. Excitingly, our data revealed significant depletion in the levels of D-sedoheptulose-7-phosphate under all treatment conditions (Fig. 3). As D-sedoheptulose-7-phosphate is also a key early precursor metabolite in the heptose biosynthesis pathway, our data suggest that colistin, doripenem, and their combination perturb the biosynthesis of ADP-heptose in *A. baumannii* via inhibition of the PPP. Another metabolite in the PPP, D-ribose 5-phosphate, was depleted after treatment with colistin, doripenem and the combination (Fig. 3). D-Ribose 5-phosphate is a key initial precursor metabolite in purine and pyrimidine metabolism, and hence all treatment conditions caused significant decreases in the levels of nucleotides, both purine and pyrimidine (Fig. 4B). Previous metabolomics studies have shown total depletion of the nucleotide pool following antibiotic treatment (ampicillin, kanamycin, norfloxacin, and vancomycin) in both Gram-negative (*Escherichia coli*) and Gram-positive (*Staphylococcus aureus*) bacteria^40^,^58^. The significant changes in nucleotide levels in antibiotic-treated samples were suggestive of nucleotide degradation^40^. Interestingly, significant depletion in the levels of nucleotides in the polymyxin-resistant LPS-deficient strain *A. baumannii* 19606 R was observed even without polymyxin treatment^53^. Significant depletion in the levels of ATP, NADP^+^ and NAD^+^ is likely secondary to the nucleotide pool depletion, but may also be indicative of altered oxidative phosphorylation. It has been reported that polymyxins induce inhibition of respiration which reduces the level of the intracellular ATP pool^59^, and altered levels of TCA metabolites (fumarate and *cis*-aconitate) were observed in the present study. It is likely that the depletion of energy related metabolites by colistin, doripenem and the combination is a secondary effect of their antibacterial activity against *A. baumannii*.

The broad-spectrum antibacterial effect of doripenem against Gram-positive and Gram-negative bacteria is by virtue of its ability to inhibit biosynthesis of the key building block of the bacterial cell wall, peptidoglycan^39^,^60^,^61^. Fundamentally, doripenem is a substrate analogue that binds to the *C*-terminal transpeptidase active site of PBPs in a non-reversible manner, thus inhibiting the peptidoglycan polymerization process^62^. Notably, following treatment with doripenem alone or in combination at 4 hr, we observed a significant decrease in the levels of the peptidoglycan biosynthesis metabolites, meso-2,6-diaminoheptanedioate and UDP-*N*-acetylmuramoyl-L-alanyl-D-glutamyl-6-carboxy-L-lysyl-D-alanyl-D-alanine (Fig. 5B). As mentioned above, colistin monotherapy also significantly decreased the levels of the essential peptidoglycan precursor metabolites UDP-GlcNAc and UDP-MurNAc (Fig. 5A). Interestingly, our previous transcriptomics results showed that peptidoglycan-associated lipoproteins were significantly up-regulated in *A. baumannii* in response to treatment with colistin and doripenem alone or in combination^41^. The up-regulation of peptidoglycan-associated lipoproteins may be a protective action by *A. baumannii* to cope with the inhibition of peptidoglycan synthesis by doripenem and/or colistin. Taken together, our current metabolomic study reveals that, in addition to disorganizing the OM, colistin also inteferes cell wall synthesis via inhbition of peptidoglycan metabolism; this mechanism also explains the synergistic killing effect of its combination with a carbapenem.

Studies have shown that the mechanism of polymyxin activity was partly associated with oxidative stress via the formation of hydroxyl radicals, with reactive oxygen species mainly targeting DNA, RNA, proteins and lipids^63^, or by inhibition of respiratory chain enzymes (e.g. NADH-quinone oxidoreductase)^64^,^65^. However, the association of free radicals in the mechanism of antibiotic bacterial killing is disputable^66^,^67^,^68^,^69^,^70^. In our analysis the reduced form of glutathione (GSH), an important indicator of oxidative stress, was not detected, as it was likely oxidized to glutathione disulfide (GSSG) during sample preparation and/or storage^71^. Nevertheless, the total glutathione content, measured as GSSG, was significantly depleted following exposure to colistin and doripenem alone and in combination (Supplementary Table 1); this result is in line with the utilization of glutathione pools to compensate for antibiotic-induced oxidative damage, albeit not consistent with the increased levels of reduced glutathione previously reported^40^. Even though we were unable to detect specific markers of oxidative stress from the TCA cycle intermediate (i.e. α-ketoglutarate) and product (i.e. NADH), the changes to other TCA metabolites (i.e. fumarate and *cis*-aconitate) clearly indicate the perturbation of the TCA cycle in response to single and combination treatments of colistin and doripenem. Our group previously demonstrated that *A. baumannii* ATCC 19606 treated with colistin significantly increased the expression of superoxide dismutase (SOD) enzyme, HMPREF0010_02336 (*sodB* encoding a predicted FeSOD) and HMPREF0010_02564 (encoding a predicted Cu-ZnSOD), suggesting the association of hydroxyl radicals in colistin antibacterial activity^41^.

To the best of our knowledge, this is the first metabolomics study to investigate the mechanism of action of colistin either as monotherapy, or in combination with doripenem, against *A. baumannii*. Our study discovered significant perturbations to cell envelope biosynthesis, nucleotide metabolism, and energy metabolism by colistin and its synergistic combination with doripenem. The convergence of antibiotic-induced metabolic profiles on the depletion of PPP and amino-sugar metabolites indicates that these pathways play key roles in the antibacterial activity of colistin alone and its combination with doripenem. Importantly, we are the first to demonstrate that the combination of colistin with doripenem synergistically kills *A. baumannii* via inhibiting different key metabolic pathways in a time-dependent manner, which highlights the essentiality of mechanism-based optimization of this combination using pharmacokinetics/pharmacodynamics. Overall, this study highlights the importance of elucidating the complex and dynamic interaction of multiple cellular metabolic pathways due to antibiotic treatment, and the significant potential of systems pharmacology in paradigm-shifting optimization of antibiotic use in patients.

## Materials and Methods

### Strain, antibiotics and reagents

*A. baumannii* ATCC 19606 (American Type Culture Collection [ATCC], Manassas, USA) was susceptible to both colistin and doripenem with MICs of 1 mg/L for both antibiotics. The strain was grown in cation-adjusted Mueller-Hinton broth (MHB; Oxoid, Australia; 20–25 mg/L Ca^2+^ and 10–12.5 mg/L Mg^2+^). Colistin (Sigma-Aldrich, Saint Louis, USA) and doripenem (Doribax, Shinogi Inc, Osaka, Japan) were prepared using Milli-Q water (Millipore Australia, North Ryde, New South Wales, Australia) prior to each experiment and sterilized by filtration with a 0.22-μm pore size Millex GP filter (Millipore, Bedford, MA).

### Bacterial culture preparation

Culture of *A. baumannii* ATCC 19606 was prepared on a nutrient agar plate from the frozen stock (−80 °C) and incubated for 16–18 hr at 37 °C. For the overnight culture, a colony of ATCC 19606 was inoculated into 15 mL MHB and incubated for 16–18 hr at 37 °C with shaking at 150 rpm. For the main culture, 1:100 dilution of the overnight culture was sub-cultured into four different reservoirs containing 200 mL fresh MHB and grown to an optical density at 600 nm (OD_600_) of ~0.5 to achieve the starting inoculum ~10^8^ cfu/mL (in order to obtain enough cells) of an early exponential growth phase. Bacterial culture was treated with colistin (2 mg/L), doripenem (25 mg/L), and combination of colistin and doripenem (2 mg/L + 25 mg/L, respectively); concentrations of colistin and doripenem were chosen based on their pharmacokinetics in patients^26^. Bacterial culture without any antibiotic treatment served as a control. Four biological replicates were prepared independently from different colonies of ATCC 19606 on different days.

### Preparation of cellular metabolite extracts

The untargeted metabolomics study was performed to investigate global metabolic alterations in *A. baumannii* ATCC 19606 due to colistin, doripenem and the combination treatments in an *in vitro* static time-kill study. Cellular metabolites of *A. baumannii* were extracted by the previously optimized method with slight modifications^53^. Samples were collected before treatment with colistin, doripenem and the combination (i.e. time = 0), and at 15 min, 1 hr, and 4 hr for metabolite extraction and viable counting. For the fingerprint samples (i.e. intracellular metabolites), 15 mL of the bacterial culture was collected and immediately transferred on ice. All the samples were rapidly quenched in a dry ice/ethanol bath and preserved on ice for all following steps. Samples were normalized by optical density (OD_600_ _nm_) and centrifuged for 10 min at 3,220 *g* at 4 °C. The supernatant was collected for extracellular metabolites (i.e. footprint). The cell pellets were washed three times with sterile saline (4 °C) and centrifuged for 3 min at 3,220 *g* at 4 °C. Cellular metabolites were extracted with chloroform:methanol:water (1:3:1, v/v; −80 °C) (total volume of 300 μL) containing generic internal standards (CHAPS, CAPS, PIPES and TRIS) at 1 μM. Samples were immediately frozen in liquid nitrogen and allowed to thaw on ice, and the freeze-thaw process was repeated three times to lyse the cells and release cellular metabolites. The extracted samples were centrifuged for 10 min at 3,220 *g* at 4 °C and the supernatant was collected and further centrifuged at 14,000 *g* for 10 min at 4 °C. The final supernatant samples (200 μL) were collected into injector vials for LC-MS analysis. For footprint samples, aliquots of the culture supernatant were rapidly filtered through a 0.22-μm membrane filter, and 10 μL of the supernatant was mixed with 250 μL of chloroform:methanol:water (1:3:1, v/v) and centrifuged at 14,000 *g* for 10 min at 4 °C to collect particle-free supernatant for LC-MS analysis.

### LC-MS analysis of metabolites

Samples were analyzed on a Q-Exactive Orbitrap mass spectrometer (Thermo Fisher), coupled to a Dionex high-performance liquid chromatograph (U3000 RSLC HPLC, Thermo Fisher) with a ZIC-pHILIC column (5 μm, polymeric, 150 × 4.6 mm; SeQuant, Merck). The MS system was operated at 35,000 resolution in both positive and negative electro-spray ionization (ESI) mode (rapid switching) and a detection range of 85 to 1,275 *m/z*. The LC solvent consisted of 20 mM ammonium carbonate (A) and acetonitrile (B) with a multi-step gradient system from 80% B to 50% B over 15 min, then to 5% B at 18 min, followed by a wash with 5% B for 3 min, and re-equilibration for 8 min with 80% B at a flow rate of 0.3 mL/min^53^. The injection sample volume was 10 μL and the run time was 32 min. All samples were analyzed in the same run and the chromatographic peaks, signal reproducibility and analyte stability were monitored by assessment of pooled biological quality control (PBQC) samples (aliquot of 10 μL of each sample, including both footprints and fingerprints) analyzed periodically throughout the batch, internal standards and total ion chromatograms for each sample. Mixtures of pure standards containing over 200 metabolites were analyzed within the batch to aid in the identification of metabolites.

### Data processing, bioinformatics and statistical analyses

Metabolomics data analyses were performed as previously described^53^ using mzMatch^72^ and IDEOM (http://mzmatch.sourceforge.net/ideom.php)^73^. Quantification of each metabolite was conducted using the raw peak height. Univariate and multivariate analyses utilized MetaboAnalyst 3.0^74^. Prior to analysis, relative peak intensity data were normalized by the median, log transformed and scaled (by auto scale function) to reduce variance between the samples. The global metabolic profiles of samples with antibiotic treatments at each time point were analyzed using multivariate statistical analysis by unsupervised principal component analysis (PCA). One-way Analysis of Variance (ANOVA) (*p* < 0.05, FDR ≤ 0.1) for multiple comparison and post hoc analysis using Tukey’s Honestly Significant Difference (Tukey’s HSD) were applied to identify significant metabolite changes between treated and untreated control samples at each time point. Metabolites that were detected as isomeric peaks with opposite abundance changes (increased and decreased levels) were excluded. To further increase the reliability of the data, significant metabolites were filtered by selection of only those that showed a ≥ 1.5-log_2_-fold change relative to the untreated control samples and an identification confidence score of 6 or more in IDEOM (i.e. removing likely LC-MS artefacts). Metabolic pathway analysis was performed based on the statistically significant identified metabolites (≥1.5-log_2_-fold; *p* ≤ 0.05, FDR ≤ 0.1, one-way ANOVA for multiple comparison). Visualization and Analysis of Networks containing Experimental Data (Vanted) software was utilized to visualize the associated metabolic pathways^75^.

## Additional Information

**How to cite this article:** Maifiah, M. H. M. *et al*. Untargeted metabolomics analysis reveals key pathways responsible for the synergistic killing of colistin and doripenem combination against *Acinetobacter baumannii. Sci. Rep.* **7**, 45527; doi: 10.1038/srep45527 (2017).

**Publisher's note:** Springer Nature remains neutral with regard to jurisdictional claims in published maps and institutional affiliations.

## Supplementary Material

Supplementary Information

Supplementary Table 1

Supplementary Table 2
