# Supplementary material for: Untargeted metabolomics analysis reveals key pathways responsible for the synergistic killing of colistin and doripenem combination against Acinetobacter baumannii
Source: Sci Rep. 2017 Mar 30;7:45527. doi: 10.1038/srep45527 (PMC5371981; doi:10.1038/srep45527)
Supplement: Supplementary Information [file srep45527-s1.pdf]

Untargeted metabolomics analysis reveals key pathways responsible for the synergistic killing of colistin and doripenem combination against *Acinetobacter baumannii*

Mohd Hafidz Mahamad Maifiah[1], Darren J. Creek[1,†], Roger L. Nation[1], Alan Forrest[2], Brian T. Tsuji[3], Tony Velkov[1], Jian Li[4,†,*]

[1]Drug Delivery, Disposition and Dynamics, Monash Institute of Pharmaceutical Sciences, Monash University, Parkville, VIC, 3052, Australia.

[2]UNC Eshelman School of Pharmacy, The University of North Carolina at Chapel Hill, Chapel Hill, NC, 27599-7569, USA.

[3]Department of Pharmacy Practice, University at Buffalo, Buffalo, NY, USA.

[4]Biomedicine Discovery Institute, Department of Microbiology, Monash University VIC 3800, Australia.

†Joint senior authors: Jian Li, email: Colistin.Polymyxin@gmail.com, phone: +61 3 9903 9702, fax: +61 3 9902 9222; Darren J. Creek, email: darren.creek@monash.edu, phone: +61 3 9903 9249, fax: +61 3 9903 9583.

*Corresponding author: Jian Li, email: Colistin.Polymyxin@gmail.com

Supplementary Information

(A)

	Median RSD (%)
**15 min**	
Control	18
Colistin	28
Doripenem	19
Combo	30
**1 hr**	
Control	18
Colistin	23
Doripenem	22
Combo	19
**4 hr**	
Control	26
Colistin	24
Doripenem	29
Combo	21
PBQCs	14

(B)

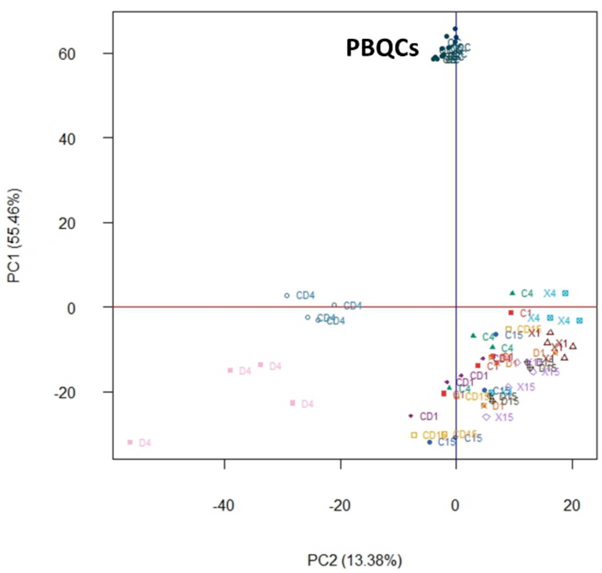

Supplementary Figure S1. (A) Data precision of individual samples represented as the median relative standard deviation (RSD) for all metabolites based on all replicates (n=4) of each group (n=11 for technical replicates of PBQCs). (B) PCA score plot of all metabolites of *A. baumannii* ATCC 19606 treated with colistin and doripenem alone and in combination. Eleven PBQCs were analyzed throughout the LC-MS batch. Each dataset represents four biological replicates of antibiotic-treated and untreated control samples of all the time points. Pooled biological quality controls = PBQCs.

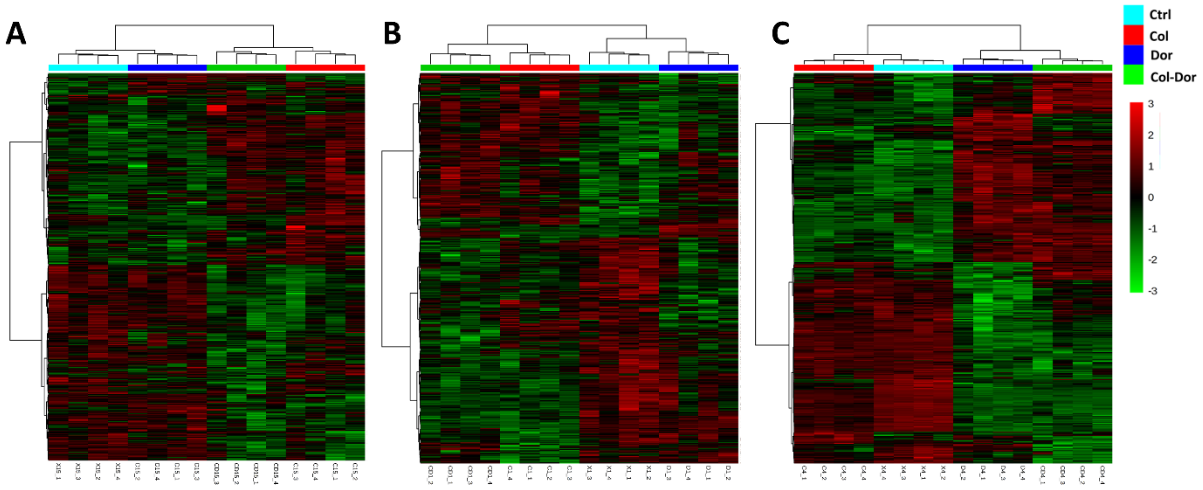

Supplementary Figure S2. Monotherapy and combination of colistin and doripenem induce global metabolic changes. Heatmap profiles with hierarchical clustering of top 600 significantly changed metabolites after treatment with single and combination of colistin and doripenem at **(A)** 15 min, **(B)** 1 hr, and **(C)** 4 hr. Light blue = untreated control (Ctrl); Red = Colistin (Col); Blue = Doripenem (Dor); Green = colistin and doripenem combination (Col-Dor). $p \leq 0.05$, FDR ≤ 0.1.

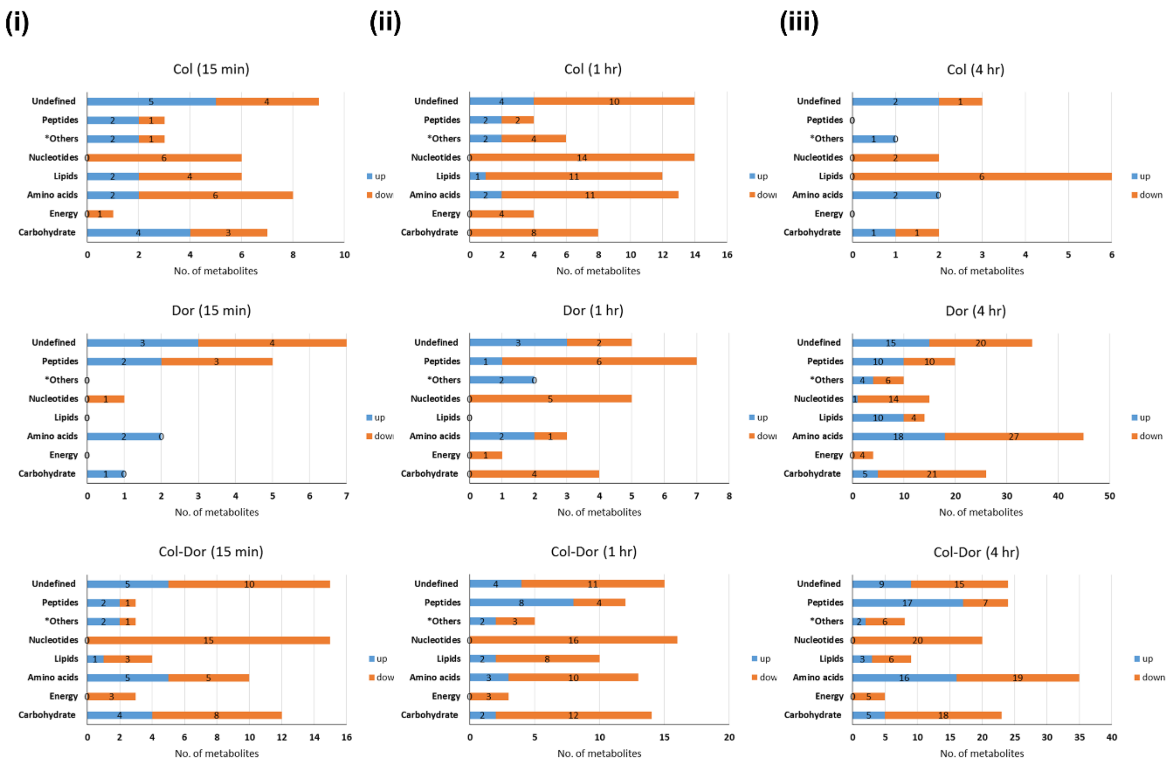

Supplementary Figure S3. Summary number of metabolites changes classified according to different metabolite classes after antibiotic treatment at (i) 15 min, (ii) 1 hr, and (iii) 4 hr. Changes ≥ 1.5-log$_2$-fold, $p \leq 0.05$, FDR ≤ 0.1 (one-way ANOVA for multiple comparison).
